# Social Influences on Early Fairness Expectations in Toddlers: Siblings, Peers, and Caregivers

**DOI:** 10.1111/infa.70055

**Published:** 2025-11-28

**Authors:** Marek Meristo, Sara Szepanski, Karin Strid

**Affiliations:** ^1^ Department of Psychology University of Gothenburg Gothenburg Sweden

**Keywords:** fairness expectations, infant development, mental state language, parent‐child interaction, preschool attendance, sibling influence

## Abstract

This study examined how social factors such as parental mental state language, sibling presence, and preschool attendance influence their expectations of fairness in 18‐month‐old toddlers. Fifty‐four toddlers participated in a nonverbal fairness task and a picture‐description task with their caregivers. We also collected questionnaire data concerning the presence of siblings and preschool attendance. The fairness task involved an animated scenario with a mouse distributing bananas to monkeys, measuring the infants' attention to equal and unequal outcomes. The picture‐description task evaluated how caregivers used mental state language when discussing morally relevant illustrations with their toddlers. We found no significant link between parents' use of mental state language and toddlers' fairness expectations. Similarly, having siblings did not appear to influence these expectations. In contrast, toddlers who attended preschool demonstrated increased sensitivity to unfair outcomes. These results highlight the importance of early social interactions involving peers in preschool environments in the development of fairness expectations in toddlers.

## 1 Introduction

Understanding the development of moral principles in humans, especially regarding notions of fairness, is essential for predicting and possibly shaping individual well‐being and societal dynamics. Recent studies using nonverbal violation‐of‐expectation looking time measures, where longer looking indicates surprise at an unexpected event, provide compelling evidence that infants develop sensitivity to fairness principles remarkably early. Such sensitivity has been observed in infants who detect violations to 1:1 resource allocation as early 4 and 9 months (Buyukozer Dawkins et al. 2019), 10 months (Meristo et al. 2016; Meristo and Surian 2013), 12 months (Ziv and Sommerville 2017), as well as later in the second year at 15 months (Schmidt and Sommerville 2011) and 19–21 months (Sloane et al. 2012). Furthermore, infants' expectations of fairness appears to be more nuanced than a simple grasp of 1:1 equality, as they demonstrate an ability to consider context‐sensitive information. Nineteen‐month‐olds are able to perceive an equal distribution as unexpected when individuals differ in their work effort and deservingness (Sloane et al. 2012), and 17‐month‐olds expect distributors to exhibit favoritism toward members of their own group when resources are scarce (Bian et al. 2018). Additionally, other findings not only reveal that infants can detect fairness violations but also indicate their ability to assign positive or negative evaluations to the individuals responsible for those violations (Meristo and Surian 2014; Surian et al. 2018; Surian and Franchin 2017). In one study, ten‐month‐old infants looked longer when antisocial actions (such as taking away a toy or hitting) were directed toward a previously unfair distributor rather than a fair one, indicating that infants expect unfair individuals to be more likely targets of punishment (Meristo and Surian 2014). However, note that longer looking times in violation‐of‐expectation paradigms do not always indicate surprise at unexpected events. The interpretation of looking patterns in socio‐moral contexts remains complex, as some studies show longer looking at expectation‐violating events while others find longer looking at expectation‐consistent events, depending on contextual factors. Finally, infants surpass a mere registration of distribution outcomes, actively considering the intentions of the distributors whose actions generate those outcomes (Geraci et al. 2022; Strid and Meristo 2020; Woo et al. 2023). For instance, ten‐month‐old infants in Strid and Meristo (2020) showed longer looking times when a third agent approached a distributor who attempted but failed to distribute resources unequally compared to one who attempted but failed to distribute equally. Collectively, these findings from looking‐time measures reveal sophisticated fairness expectations emerging before children can verbally articulate moral judgments.

Importantly, recent cross‐cultural research suggests that these early fairness expectations might be influenced by cultural context from early infancy. Meristo and Zeidler (2022) compared 12‐ to 20‐month‐old infants from Sweden with infants from Samburu and Kikuyu populations in Kenya using a violation‐of‐expectation paradigm similar to previous studies. While Swedish infants showed the typical Western pattern of looking longer at unequal distributions (suggesting an expectation of equality), Samburu infants demonstrated the opposite pattern, expecting unequal distributions and looking significantly longer when resources were distributed equally. Kikuyu infants showed no specific expectations, looking equally at both conditions. These findings suggest that cultural experiences might shape fairness expectations even before the second year of life, highlighting the importance of examining social and environmental factors that contribute to early moral development.

While recent research has made significant advances in understanding infants' sensitivity to fairness, we know surprisingly little about which social factors influence individual differences in these emerging intuitions. Most studies have examined fairness expectations in isolation from children's social environments, leaving a critical gap in our understanding of how early fairness expectations develop within social contexts. This study addresses this gap by simultaneously investigating three potential influences on 18‐month‐old toddlers' fairness expectations: parental mental state language during moral discussions, sibling relationships, and preschool attendance. By using a nonverbal looking‐time fairness task with toddlers, we explore whether their expectations are primarily shaped by language‐rich familial interactions or by direct experiences with siblings and peers at preschool.

This study investigates early fairness expectations, defined as implicit, nonverbal responses to distributive justice scenarios, measured through looking‐time patterns (Sloane et al. 2012). These expectations represent a foundational component of moral development, distinct from explicit verbal moral reasoning, conscious deliberation about right/wrong; or prosocial behavior, that is actions intended to benefit others (Dahl and Paulus 2019). While older children's moral judgments involve explicit verbal evaluations of actions as morally right/wrong (Killen and Smetana 2015), infants' looking‐time responses reflect surprise at norm violations, indicating an emerging sensitivity to fairness norms (Schmidt and Sommerville 2011). The question of whether implicit fairness expectations and prosocial behavior constitute moral understanding remains unresolved (Buyukozer Dawkins, Ting, et al. 2019; Dahl and Brownell 2019; Dahl and Paulus 2019; Hoffman 2007).

## 2 Role of Mental State Language

There is by now substantial evidence for a link between parents' use of mental state language and children's development of theory of mind (ToM) (Devine and Hughes 2018; Ruffman 2023; Tompkins et al. 2018). For instance, Ruffman et al. (2002) discuss the importance of mental state talk, which involves conversations and discussions about emotions, thoughts, and beliefs, in fostering children's ability to attribute mental states to themselves and others. The authors highlight that through mental state talk, children learn to navigate social interactions, interpret others' behavior, and make predictions about their intentions. These conversations provide a foundation for children to develop a deeper understanding of the inner lives of individuals, leading to the construction of a theory of mind. Some limited support for the relation between false belief and mental state talk has been found even among infants and their caregivers (Roby and Scott 2018).

Experimental evidence further supports the causal role of mental state language in shaping social cognition. In an intervention study, Grazzani et al. (2016) demonstrated that structured conversations with adults about mental states significantly enhanced social understanding in toddlers. Over a 1‐month intervention, 2‐year‐old children participated in group discussions with adults about characters' thoughts and emotions after listening to illustrated stories. Compared to a control group that discussed physical events, the experimental group showed marked improvements on theory of mind and emotion understanding measures.

While most of the research has focused on the link between parent mental state talk and children's false‐belief understanding, there is a theoretical basis for expecting a relationship with moral understanding as well. Moral understanding involves evaluating the rightness of actions based on understanding of others' mental states, such as their intentions, beliefs, and desires, to evaluate the permissibility of actions. In other words, theory of mind plays a crucial role in children's moral evaluations, as it equips them with the ability to identify, understand, and respond to the mental states of others in a morally sensitive manner (Ball et al. 2017; Buon et al. 2016; Decety et al. 2012; Du et al. 2024; Forman et al. 2004; Lagattuta and Kramer 2022; Sodian et al. 2016; Young et al. 2007). Parents who engage their children in discussions about mental states thereby enhance their children's capacity to recognize and consider these states in others, which may thereby foster the growth of moral understanding. Research also indicates that parental mental state language contributes to children's prosocial behaviors (Brownell et al. 2013; Drummond et al. 2014; Grazzani, Ornaghi, Agliati, et al. 2016), which are theoretically linked to moral understanding and reasoning.

The use of mental state language may be theoretically integral also to children's understanding of fairness. In fairness situations, such language may help children comprehend and articulate the perspectives of others involved in resource distribution. For example, when discussing fairness, caregivers might express desires (She wants the same amount as her), beliefs/thoughts/cognitions (He thinks it's not fair), or emotions (They feel sad about getting less). Caregivers and educators could employ mental state language to scaffold children's understanding of fairness, using phrases such as “How do you think she feels about getting fewer stickers?” or “Do you believe he wanted an equal share?” By incorporating mental state language into discussions about fairness, children might develop a more nuanced understanding of fairness concepts.

There is also longitudinal evidence suggesting that toddlers are sensitive to mental state terms. A study by Taumoepeau and Ruffman (2006) examined the relationship between mothers' use of mental state language and their 15‐24‐month‐old children's development of desire language and emotion understanding. The researchers found that mothers' use of desire language with 15‐month‐olds uniquely predicted children's mental state language and emotion task performance at 24 months, even after controlling for confounding variables. This predictive relationship suggests that toddlers process parental mental state talk, even if they cannot yet demonstrate full comprehension. They also found that mothers decreased their use of desire language over time while increasing references to thoughts and knowledge. The relationship between mother's desire talk and child's mental state language appeared unidirectional, that is early maternal desire talk predicted later child mental state language, but not vice versa.

While previous research has established robust links between parental mental state language and children's theory of mind, prosocial behavior, and emotion understanding (e.g., Brownell et al. 2013; Drummond et al. 2014; Taumoepeau and Ruffman 2006; Tompkins et al. 2018), much less is known about whether and how this form of parental input shapes the development of fairness expectations in infancy. Fairness understanding requires children to consider not only outcomes but also the intentions, desires, and emotions of others involved in distributive situations (Ball et al. 2017; Lagattuta and Kramer 2022; Sodian et al. 2016). Therefore, we hypothesized that toddlers whose parents more frequently use mental state language in morally relevant contexts may be better equipped to attend to and interpret the social and intentional cues that underlie fair and unfair distributions. By directly assessing the relationship between parental mental state talk during moral conversations and toddlers' nonverbal responses to fairness violations, our study extends prior work in understanding the socialization of early moral cognition.

## 3 Preschool Peers and Siblings

Parent, peer and sibling interactions provide crucial environments for the development of children's moral skills (Mammen and Paulus 2023). In interactions with parents, children benefit from an asymmetrical relationship where parents guide and scaffold their understanding of moral norms and prosocial behaviors (Killen and Smetana 2015). This dynamic allows children to acquire a foundational understanding of right and wrong within the safety of a hierarchical relationship. Conversely, interactions with peers and siblings present a different but equally important context. Here, children operate on a more equal footing, engaging in spontaneous conflicts over toys, observing each other's emotional reactions to distribution outcomes, and experiencing direct consequences of fair and unfair exchanges (Dunn 2002; Johansson 2002). This peer and sibling interaction fosters a sense of justice, fairness, and the ability to see multiple perspectives, which are critical components of moral development (Piaget 1932).

### 3.1 Preschool Context

Preschool or nonparental early childcare environments offer socialization contexts that may be particularly influential for the development of fairness expectations. Unlike informal peer interactions, preschool settings provide systematic exposure to distributive situations where children observe and participate in resource allocation under structured guidance (Johansson 2002). Teachers actively scaffold fairness concepts through daily activities involving sharing materials, taking turns, and resolving conflicts over resources (Johansson and White 2011). Within preschool environments, caregivers function as behavioral models while actively facilitating positive peer interactions such as sharing and cooperation, simultaneously reinforcing prosocial behaviors including helping and comforting others (Eisenberg et al. 2015). These structured interactions create repeated opportunities for children to witness and internalize equality norms before they can verbally articulate fairness principles.

The theoretical foundations for expecting preschool effects on fairness development rests on various core mechanisms. First, preschool environments provide intensive peer exposure during a critical developmental window when early fairness expectations are emerging (e.g., Ziv and Sommerville 2017). Children observe multiple distributive events daily, from snack time to toy sharing, allowing them to extract statistical regularities about how resources are be allocated. Second, teacher‐mediated interactions explicitly model fairness through verbal instructions and conflict resolution, providing linguistic scaffolding for abstract fairness concepts (Johansson and White 2011). Third, the emotional consequences of unfair treatment become salient through peer reactions, helping children understand that unequal distributions violate social expectations and cause distress (Kim et al. 2023).

Research demonstrates a significant association between early preschool attendance and children's moral and prosocial development. Brownell and Drummond (2020) found that high‐quality early childhood education characterized by warm and sensitive caregiver‐child interactions predicted enhanced prosocial behavior in first grade. These effects were robust across different types and amounts of care, indicating that the quality of preschool experiences was more influential than the specific setting or number of hours attended. Structural features such as group size and caregiver training contributed to prosocial outcomes primarily by enabling higher preschool quality which directly fostered prosocial development.

Given this theoretical foundation and empirical evidence, preschool attendance represents a promising predictor of individual differences in fairness expectations among 18‐month‐olds. The intensive peer interaction, structured resource sharing, and explicit fairness modeling may enhance sensitivity to unequal distributions even before advanced language development.

### 3.2 Sibling Relationships

Sibling relationships represent an influential socialization context in the development of early moral understanding that is distinct from both parent‐child and peer dynamics. Siblings are often children's most consistent social partners, engaging in frequent and emotionally charged interactions that provide rich opportunities for learning about fairness and justice (Dunn 2002; Howe et al. 2022; Ross 1996). Daily negotiations over shared resources create complex social situations where siblings must decide how to divide toys, take turns, or resolve disputes. These everyday encounters require children to consider both their own interests and those of others serving as natural places for practicing perspective‐taking, empathy, and conflict resolution which are foundational for the emergence of fairness expectations.

The dynamics of sibling relationships vary widely offering different pathways for moral and prosocial learning. A child's place in the sibling pair, that is whether they are older or younger, shapes the chances and expectations for showing helpful, kind actions. Older siblings tend to display more prosocial behaviors toward their younger siblings, while younger siblings often respond by following their older siblings' lead and imitating their actions (see Eisenberg et al. 2015). Additionally, close‐aged siblings are more likely to engage in egalitarian, reciprocal exchanges, which may foster mutual understanding and the development of fairness norms through shared experiences and negotiation (McAlister and Peterson 2013; Prime et al. 2014). In contrast, relationships with older siblings often involve hierarchical structures, where older children take on teaching or caregiving roles, modeling and scaffolding moral behaviors for their younger siblings. For instance, Jambon et al. (2019) report that older siblings have a stronger positive influence on their younger siblings' empathy development when there is a larger age gap between them, but the age gap doesn't affect how much younger siblings influence their older siblings' empathy.

The influence of siblings on moral development is not limited to overt acts of sharing or fairness. Through repeated exposure to their siblings' emotions, intentions, and reactions, children learn about the mental states of others, which supports the development of theory of mind and more nuanced moral understanding (Dunn 2002; McAlister and Peterson 2013). Sibling interactions also provide a context for learning about the consequences of moral and immoral actions, as children observe and experience praise, blame, or reconciliation following conflicts or acts of kindness. Thus, sibling relationships offer a rich and multifaceted environment for the early development of fairness expectations and broader moral understanding.

Finally, Ziv and Sommerville (2017) investigated developmental differences in infants' fairness expectations between the ages of 6–15 months to shed light on how these expectations change during this very early period of early development. Their findings indicate a developmental shift in the acquisition of fairness expectations that is associated with experiences of sharing, and particularly by having siblings. Infants who had siblings demonstrated a heightened attention to unfair outcomes compared to unequal outcomes, whereas infants without siblings did not show this distinction.

## 4 Present Research

Our experimental approach was informed by recent research on cultural variation in early fairness expectations (Meristo and Zeidler 2022), leading us to examine how specific social factors within Swedish culture such as preschool attendance, sibling presence, and parental mental state language might influence toddlers' developing sense of fairness. Specifically, the present research addressed three questions: (1) Does parental use of mental state language during moral discussions predict toddlers' expectations of fairness, as measured by their responses to a nonverbal resource distribution task? (2) Is the presence of siblings associated with toddlers' fairness expectations? (3) Is attendance at preschool associated with toddlers' fairness expectations? By investigating these questions, we aimed to identify which aspects of the early social environment are influential in shaping the development of fairness expectations in infancy. While sibling and peer relationships can vary widely in quality, the present study focuses on the presence and number of siblings and preschool attendance as initial measures of their potential influence on fairness expectations in toddlers.

## 5 Materials and Methods

### 5.1 Participants

We initially recruited 55 infants using a maximally inclusive strategy. Recruitment criteria were: (1) the infant's age within ± 1 month of 18 months (i.e., 17–19 months), and (2) no known issues that would directly impede the infant's ability to process visual stimuli. After testing, infants were included in the statistical analyses based on predetermined inclusion criteria established before data collection: (1) no known developmental disorders (*n* = 0), and (2) at least one caregiver spoke Swedish as their primary language of communication with the child at home (*n* = 1). Infants were also excluded for session‐level errors, including experimenter error (*n* = 0), equipment failure (*n* = 0), parental interference (*n* = 0), or infant crying (*n* = 1 excluded from the picture‐description task). For fairness tasks, infants were excluded if they looked at the outcome images for less than 2 consecutive seconds (*n* = 0).

This resulted in a final sample of 54 infants for the fairness task, and 53 infants for analyses involving the picture‐description task. For the fairness task sample (*N* = 54): *M* = 18.0 months; *Range* 17.2–19.0 months; 30 female and 24 male. Most of the children completed the picture‐description task with their mother (*N* = 40), and the remaining completed the task with their father (*N* = 13). Thirty‐four children did not have siblings, 15 children had one older sibling and 5 had two older siblings, 12 infants were attending pre‐school (for at least one month). We included two sibling‐measures: whether the infant had siblings, and the number of siblings. Although we collected data on the ages of the siblings (all of whom were older than the infants), we did not gather information on whether they share the same household. This limitation may affect our understanding of the nature of their relationship and the types of interactions they have. The caregivers' educational level was high, with 82% (*n* = 85 of 104 valid responses) holding university or college degrees and 18% (*n* = 19) having completed upper secondary education. No caregivers reported primary school as their highest education level. There were four missing responses.

Our original sample size of 55 participants was chosen to provide a reasonable balance between feasibility and statistical power. With this sample size, we achieved 0.75 power to detect medium effects (*f* = 0.25) in our hierarchical regression models with three predictors using an alpha level of 0.05. As an exploratory study in a new area without prior effect size estimates, we focused on detecting medium effects based on general guidelines.

The Swedish Ethical Review Authority reviewed and approved the study. The participants' legal guardians provided written informed consent to participate in the study. Participating families were randomly chosen from the database of the Swedish Tax Agency among families who were living in the urban area of Gothenburg, Sweden. Infants were tested at the Department of Psychology, University of Gothenburg. The data was collected between April and September 2019.

### 5.2 Research Design

We used a within‐subject fairness task with animated distributive scenarios to measure infants' looking times at equal versus unequal outcomes, adapting methodology from previous research (Meristo et al. 2016; Ziv and Sommerville 2017). Unlike prior work showing equal/unequal outcomes sequentially, we presented both simultaneously to capture individual differences in a single trial. We complemented this with a picture‐description task to quantify caregivers' mental state language during discussions of moral scenarios, and parent‐report measures of sibling presence and preschool attendance.

## 6 Materials

### 6.1 Fairness Task

Each infant sat on a parent's lap and viewed a 17‐inch monitor placed 50–70 cm away. Looking times were measured with a Tobii T120 (Tobii Technology, Sweden) near infrared eye tracker. We asked parents not to communicate with the infant during testing. Before the test trial, the infants first saw a standard 5‐point calibration procedure. Calibration was followed by presentation of the test trial.

**FIGURE 1:**
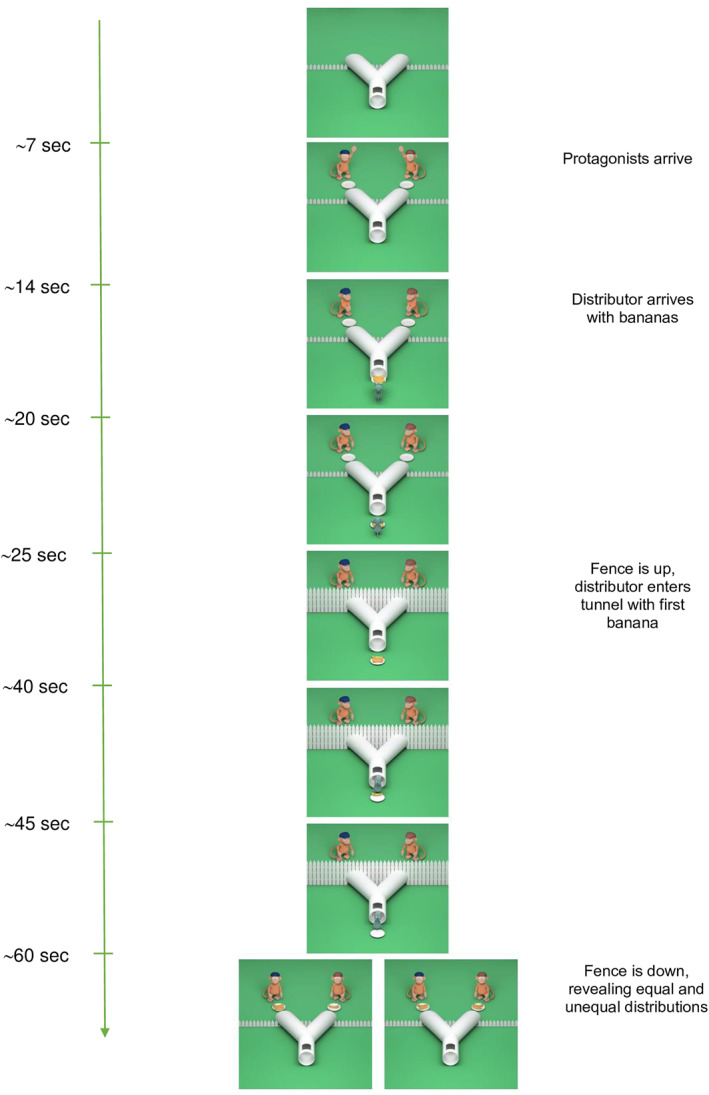
Schematic overview of the animated fairness task.

The animation began with an empty scene featuring a Y‐shaped tube with two exit points. Two monkeys entered the scene from above, each holding an empty plate (Figure 1). They walked toward separate exits of the tunnel, placed their plates down, and waved to the infants. Then, a mouse entered from below, carrying a plate with four bananas, and showed the bananas to the monkeys. Subsequently, a fence was raised in the middle of the screen, hiding the monkeys' plates. The mouse placed the plate on the ground and entered the tube four times, each time carrying a banana. After delivering the last banana, the mouse took the empty plate and left the scene. Finally, the infants saw a split screen, where a lowered fence revealed an unequal distribution (3 + 1) of bananas between the monkeys on the left side, while an equal distribution (2 + 2) was shown on the right side (the equal/unequal side counterbalanced).

Our study adapted the paradigm from Meristo et al. (2016), with two important modifications. First, in the original study by Meristo et al. (2016), infants viewed equal and unequal distributions in separate, consecutive trials. That is, they saw either an equal distribution (1:1) or an unequal distribution (2:0) in each discrete trial and their looking times to each outcome were compared between trials. In contrast, our study followed Ziv and Sommerville's (2017) approach by presenting both distribution outcomes (equal and unequal) simultaneously on a split screen at the end of the test trial. This simultaneous presentation method allows for direct assessment of infants' relative attention to equal versus unequal outcomes within the same trial which is valuable for measuring individual differences. Second, to increase infant engagement and reduce high dropout rates often observed in infant studies, we used animated 3D scenes with dynamic characters (mouse and monkeys) rather than the simple geometric shapes used in the original study.

We employed two established dependent measures for analyzing looking behavior following methodologies from prior infant studies using simultaneous outcome presentations (Meristo et al. 2012). Both measures were derived from the same eye‐tracking data collected during the final phase of the test trial when both the equal and unequal distributions were simultaneously visible to infants. For our primary, continuous measure, we calculated a differential looking score (DLS) by subtracting looking time to the equal outcome from looking time to the unequal outcome, and dividing by the sum of looking times to both outcomes. The resulting DLS values range from −1 to +1, with positive valued indicating proportionally longer looking toward the unequal outcome, negative values indicating proportionally longer looking toward the equal outcome, and zero indicating equivalent attention to both outcomes. Descriptive statistics are shown in Table 1. Analyzing the DLS is favored over comparing raw looking times at the two outcome images since the looking times are interdependent (Southgate et al. (2007)).

**TABLE 1:** Descriptive statistics for mental state talk in the picture‐description task.

	Mean	Range
Child age (months)	18.0	17.2–19.0
DLS	0.13	−0.71–0.98
Total number of words	837	310–1606
Mental state words		
Cognitive references (%)	0.90	0–2.36
Desire references (%)	1.02	0–2.65
Emotion references (%)	1.19	0–3.90
Total mental state words (%)	3.18	0.53–6.76
Total mental state words	27.19	3–77

For our secondary, categorical measure, we used the same test trial looking time data to code whether each infant looked longer at the equal or the unequal distribution. This classification allowed us to determine the proportion of infants who looked longer at the unequal versus equal outcome, which could then be compared against chance performance using a binomial test.

### 6.2 Picture‐Description Task

**FIGURE 2:**
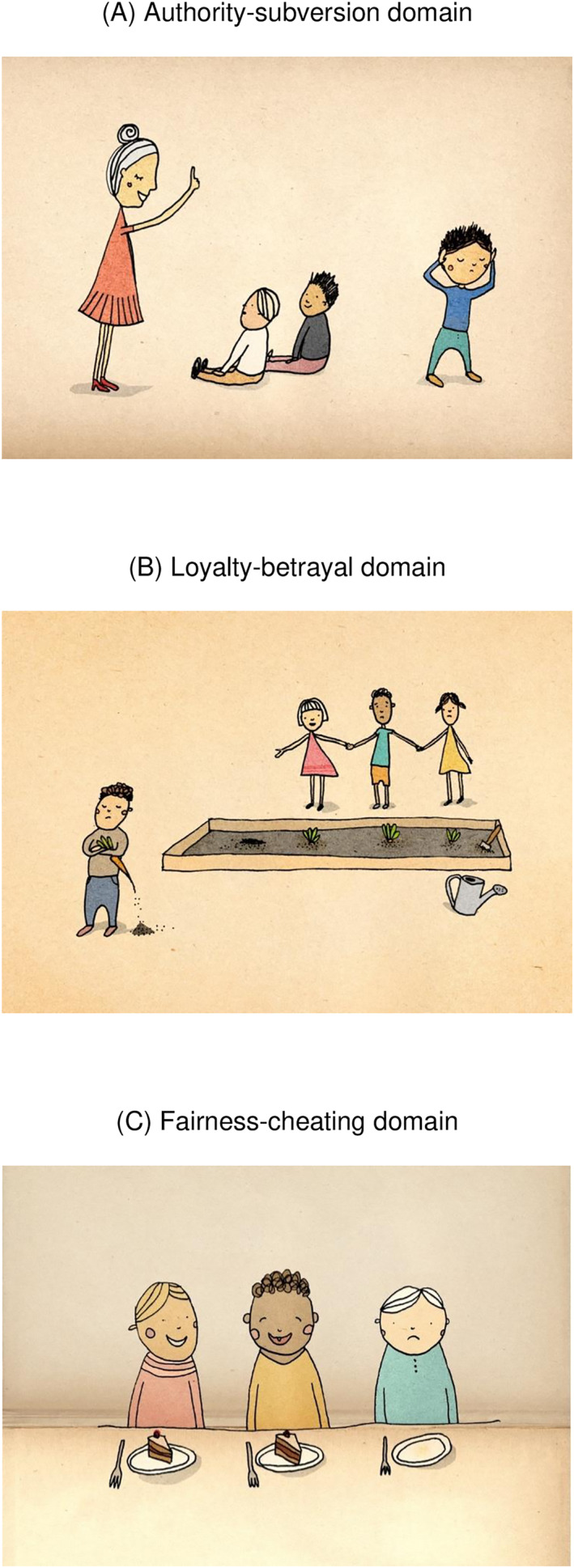
Examples of picture‐description task.

The task involved 10 illustrations created by an artist specifically for this study, drawing inspiration from the moral foundation theory as outlined by Graham et al. (2013). Moral foundations theory suggests that human moral reasoning is based on a set of universally available psychological systems. These foundations are believed to have evolved in response to adaptive challenges faced by our ancestors (Haidt and Joseph 2004). Two images depicted each of the five foundations identified by the theory, that is care/harm, fairness/cheating, loyalty/betrayal, authority/subversion, and sanctity/degradation (see Figure 2). These images portrayed various scenarios, such as one child constructing a tower while another demolishes it (illustrating care/harm), unequal cake sharing among children (depicting fairness/cheating), and acts of loyalty versus acts of disrespect.

Parents were instructed to engage in a discussion about these images with their child in a manner similar to their usual conversations at home. The illustrations were rich in moral content, offering not only scenarios for discussing mental states in different moral contexts but also everyday scenes and objects. This allowed for conversations that could extend beyond or focus on topics other than mental states. Consequently, the nature of the illustrations enabled the observation of individual variances in how parents chose to discuss mental states with their children in morally relevant scenarios. Examples of caregiver‐child conversations included:

Authority‐subversion (see Figure 2A): Parent ‐ Is he sad? Child ‐ Schaa (Sad). Parent ‐ Yeah. Maybe it’s because…What is she doing then? Child ‐ Schaaad (Sad). Parent ‐ She is standing there pointing. Maybe she is telling him off. She is saying something, and maybe he doesn’t want to join and listen.

Loyalty‐betrayal image (see Figure 2B): What is this then? They are supposed to sow together and then that one takes the carrot. Wants it for itself.

Fairness/cheating image (see Figure 2C): *Do you think its someone's birthday? Do you think that child is sad because it didn't get any cake, or because it ate its cake before the others? He can borrow your dummy when he is sad, yes?*

The caregiver‐child interactions were video‐recorded and subsequently transcribed verbatim by a trained research assistant. To ensure reliability, a second independent coder double‐checked eight randomly selected conversations (approximately 15% of the sample). Interrater agreement was high, with 95% agreement for emotion terms, 90% for cognitive terms, and 93% for desire terms. The language use of caregivers was analyzed for mental state categories using the methodology proposed by Ensor and Hughes (2008). This involved examining various aspects of caregivers' conversations that reflected their discussions or references to mental states. The categories encompassed cognitive terms, which included thoughts and knowledge, such as the use of words like “think” or “know”. Emotions including expressions such as “happy”, “pleased”, “sad”, “worried”, or “bored” were also coded. Additionally, desire categories involved references to wants, preferences, or hopes, such as the use of words like “want”, “like”, “don't like”, or “hope”. To control for parental verbosity, we calculated proportions of each type of reference in relation to total number of words used by the parent (see Table 1).

## 7 Results

### 7.1 Statistical Strategy

Our statistical analysis followed three sequential phases. First, we analyzed infants' looking behavior in the fairness task using two measures: (1) differential looking scores (DLS) comparing attention to equal versus unequal outcomes, and (2) fixation preferences (equal/unequal) using binomial tests. Second, we compared DLS‐s between groups (preschool attendance, sibling presence) with *t*‐tests and Mann‐Whitney *U* tests. Third, we explored relationships between DLS and mental state language using Pearson correlations and hierarchical regression models that controlled for siblings/preschool while testing cognition/emotion/desire terms. Mental state language measures were proportionally coded relative to total utterances to account for verbosity differences, ensuring fair comparisons across dyads.

### 7.2 Fairness Task

A one‐sample *t*‐test with the DLS as the dependent variable revealed that infants performed significantly above the chance of zero, *t* (53) = 2.91, *p* = 0.005, *d* = 0.396. The data indicated no significant differences between groups based on the infants' sex, *t* (52) = 1.54, *p* = 0.129, *d* = 0.423. Consequently, the data were pooled across this factor for subsequent analyses.

Thirty‐five out of 54 children (65%) looked longer at the unequal outcome, while the remaining 19 children (35%) looked longer at the equal outcome. This proportion differed significantly from chance (50%), *p* = 0.040, two‐tailed exact binomial test. The preference for the unequal outcome, both in terms of longer looking times and the number of children who exhibited this behavior, suggests that toddlers found the unequal outcome unexpected. This reaction could be seen as an early sign of developing a sense of fairness.

**FIGURE 3:**
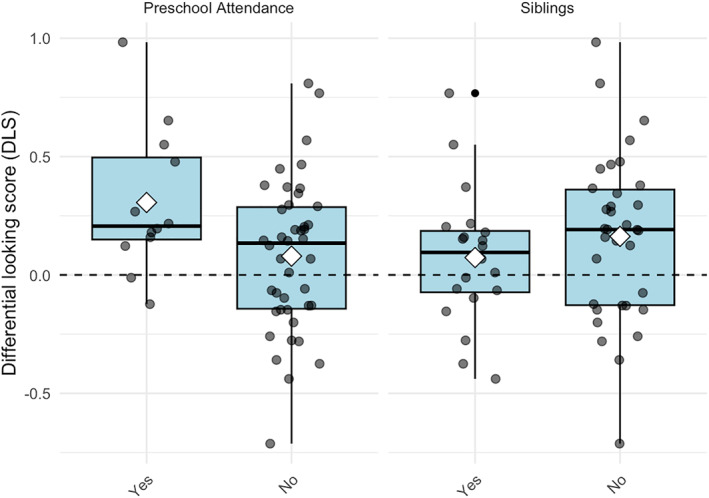
Differential looking score (DLS) across different conditions. Boxplots show the distribution of DLS scores for preschool attendance and siblings. The *x*‐axis labels indicate the categories within each condition. The boxplots display the median, first and third quartiles, and minimum and maximum values. White diamonds represent the mean DLS for each category. The horizontal dashed line at DLS = 0.0 indicates the chance level.

Independent samples *t*‐tests showed no significant effect of the presence of siblings, *t* (52) = 0.966, *p* = 0.339, *d* = 0.272. There was however a significant effect of attending preschool, *t* (52) = 2.19, *p* = 0.033, *d* = 0.715 (Mean DLS_Preschool_ = 0.31, 95% CI [0.11, 0.50]; Mean DLS_NoPreschool_ = 0.08, 95% CI [−0.02, 0.18]) (Figure 3). Children's DSL‐scores were not correlated with number of siblings, *r* = −0.16, *p* = 0.252. We also conducted a Mann‐Whitney *U* test to account for the unequal and small sample sizes. The results corroborated the findings from the *t*‐test, indicating a significant difference in DLS scores between children who attended preschool and those who did not, *U* = 347, *p* = 0.048, *r* = 0.27. The median DLS score for children who attended preschool (Mdn = 0.21) was significantly higher than for those who did not attend preschool (Mdn = 0.13). There was no significant difference in DLS scores between children with and without siblings, *U* = 271, *p* = 0.222, *r* = 0.203.

### 7.3 Relationships Between Fairness and Mental State Talk

To provide context for the subsequent analyses examining the relationship between caregivers' use of mental state language and toddlers' fairness expectations, we first compared the relative frequency of cognition, desire, and emotion terms used by caregivers during the picture‐description task (see Table 1). Paired‐samples *t*‐tests showed that caregivers referred to cognitions and desires about equally, *t* (52) = 0.929, *p* = 0.357, *d* = 0.128. There was also no difference between references to desires and emotions, *t* (52) = 1.060, *p* = 0.294, *d* = 0.146. However, there were significantly more references to emotions compared to cognitions, *t* (52) = 2.126, *p* = 0.038, *d* = 0.292.

We examined if the presence of siblings, attendance at preschool, or the sex of the infant influenced the amount of the three categories of mental state talk by caregivers, finding no significant differences (all *p*‐values > 0.91).

**FIGURE 4:**
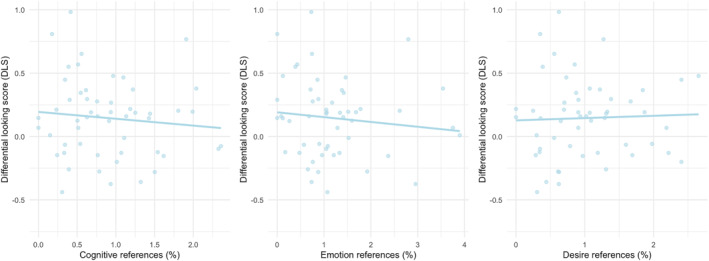
Relationship between mental state references and differential looking score (DLS) in parent‐child interactions. Each plot shows the percentage of a specific type of mental state reference on the *x*‐axis and the corresponding DLS on the *y*‐axis. The light blue dots represent individual parent‐child dyads, and the light blue trend line indicates the overall relationship between the variables.

As shown in Table 2, there were no significant associations between infants' DLS‐scores (proportional looking times in the fairness task) and caregivers' proportional use of mental state words (Figure 4). We also examined whether the raw number (rather than proportion) of mental state language terms predicted DLS scores. Neither raw counts of cognition terms (*r* = −0.10, *p* = 0.47), desire terms (*r* = 0.03, *p* = 0.83), emotion terms (*r* = −0.14, *p* = 0.33) nor total mental state words (*r* = −0.12, *p* = 0.38), showed significant associations with DLS scores.

**TABLE 2:** Correlations between infants' looking times (DLS) in the fairness task, and caregivers' use of mental state words.

	DLS	*p*
Mental state words		
Cognition	−0.10	0.46
Desire	0.04	0.79
Emotion	−0.11	0.42
Mental state words total	−0.12	0.40
Total words	−0.05	0.71

To investigate the contribution of parents' use of mental state language to children's performance on the fairness task, we conducted a series of hierarchical multiple regression analyses (see Table 3). The dependent variable was children's DLS scores on the fairness test. In the first step of each model, we entered two variables: whether the child had an older sibling, and whether the child attended preschool. These variables accounted for 13% of the variance in children's DLS scores, *F* (2, 50) = 3.75, *p* = 0.03. In the first model, we entered the percentage of utterances containing cognition terms at Step 2. This variable accounted for an additional 1.6% of the variance in children's DLS scores, *F*_change_ (1, 49) = 0.89, *p* = 0.349. That is, children who heard a higher percentage of cognition terms did not exhibit a stronger preference for the unfair location. In the second model, we entered the percentage of utterances containing emotion terms at Step 2. This variable accounted for no additional variance in children's DLS scores, *F*_change_ (1, 49) = 0.01, *p* = 0.96. Finally, we entered the percentage of utterances containing desire terms at Step 2 in the third model. This variable accounted for an additional 1% of the variance in children's DLS scores. This increase did not reach statistical significance, *F*_change_ (1, 49) = 0.08, *p* = 0.78. The results suggest that none of the categories of parents' mental state talk significantly predicted children's performance on the DLS fairness test.

**TABLE 3:** Regression analyses predicting children's DLS‐scores from caregiver mental state language.

Predictor	Model 1: Cognitive terms			Model 2: Emotion terms			Model 3: Desire terms		
	*B* (SE)	*β*	*p*	*B* (SE)	*β*	*p*	*B* (SE)	*β*	*p*
Step 1									
Constant	0.148 (0.054)		0.01	0.148 (0.054)		0.01	0.148 (0.054)		0.01
Siblings	−0.143 (0.084)	−0.226	0.10	−0.143 (0.084)	−0.226	0.10	−0.143 (0.084)	−0.226	0.10
Preschool	0.230 (0.097)	0.314	0.02	0.230 (0.097)	0.314	0.02	0.230 (0.097)	0.314	0.02
Step 2									
Cognitive terms (%)	−0.066 (0.070)	−0.126	0.35						
Emotion terms (%)				0.002 (0.047)	0.007	0.96			
Desire terms (%)							0.019 (0.066)	0.039	0.78
Model fit									
*R* ^2^	0.13			0.13			0.13		
Δ*R* ^2^ (Step 2)	0.016			0.000			0.001		
*F* (df)	3.75 (2.50)*			3.75 (2.50)*			3.75 (2.50)*		
*F* change (Step 2)	0.89 (1.49)			0.000 (1.49)			0.08 (1.49)		
*p* (*F* change)	0.35			0.96			0.78		
*N*	53			53			53		

*Note: B* = unstandardized coefficient; SE = standard error; *β* = standardized coefficient; *p* = significance level; *R* ^2^ = proportion of variance explained by variables; Δ*R* ^2^ = change in proportion of variance explained by a variable (i.e., additional variance explained by a variable). Dependent variable DLS of fairness task.

^*^ *p* < 0.05 (two‐tailed).

## 8 Discussion

This study investigated three key questions about social influences on 18‐month‐old toddlers' fairness expectations. First, we examined whether parental use of mental state language during moral discussions predicts toddlers' fairness expectations. Second, we explored whether sibling presence is associated with fairness expectations. Third, we investigated whether preschool attendance influences fairness expectations. Our findings revealed that (1) parental mental state language did not significantly predict toddlers' fairness expectations, (2) sibling presence showed no significant association with fairness responses, and (3) preschool attendance was significantly associated with heightened sensitivity to unfair outcomes. We discuss each finding in turn below.

### 8.1 Methodological Contributions

We introduced a novel, animation‐based violation‐of‐expectation task to assess fairness expectations in 18‐month‐old toddlers. Our results show that infants looked significantly longer at unequal compared to equal resource distributions, both in continuous (*p* = 0.005, *d* = 0.40) and categorical analyses (65% looked longer at the unequal outcome, *p* = 0.040). These findings indicate that the task reliably elicits differential attention to fairness violations in this age group, in line with previous research using similar paradigms (Meristo et al. 2016; Ziv and Sommerville 2017). While the validity of looking time as a direct measure of fairness reasoning remains debated, our task demonstrates robust reliability and sensitivity to early fairness expectations, supporting its utility for future studies of socio‐moral development in infancy.

### 8.2 Parental Mental State Language and Fairness Expectations

Contrary to our expectations, parental use of mental state language during the picture‐description task did not significantly predict toddlers' fairness expectations. While previous research has established a link between parents' mental state talk and children's theory of mind development (e.g., Tompkins et al. 2018), the current study suggests that this relationship may not extend to the domain of fairness expectations during the toddler years. One interpretation is that the development of fairness expectations at this age may be more strongly influenced by peer interactions and social experiences such as those encountered in preschool, than parent‐child conversations alone.

However, there are three additional possible explanations for this null finding. First, it is possible that the effects of parental mental state language on fairness expectations are lagged and only becoming apparent later in development. Prior longitudinal research indicates that parental input in infancy can predict socio‐cognitive outcomes, including perspective‐taking, at preschool age (e.g., Meins et al. 2003; Laranjo et al. 2014; Roby and Scott 2018). Thus, parental mental state language at 18 months might lay crucial groundwork for later socio‐moral understanding, but these effects may not be immediately detectable.

Second, it is also possible that the influence of parental mental state language on moral reasoning becomes more pronounced at later developmental stages, when children's cognitive abilities and language skills are more advanced. During the toddler years, children might rely more heavily on observational learning and direct experiences to form their initial concepts of fairness, with parental language playing a more significant role in refining and solidifying these concepts as children grow older (Piaget 1932; Killen and Smetana 2015).

Third, athough the task was designed to elicit moral discussions and mental state language from parents, it may not have adequately captured the richness and complexity of real‐life interactions where moral lessons are typically conveyed. Moral learning is often deeply embedded in emotional contexts and real‐world situations, where children can observe and experience the consequences of fair or unfair actions firsthand (Dahl et al. 2022; Dahl and Paulus 2019; Turiel 2015). We note also that most existing research on mental state language focuses on children older than 18 months, so our single laboratory task at 18 months may have limited power to detect subtle mental state language effects.

### 8.3 Sibling Relationships and Fairness Expectations

Interestingly, the existence of siblings did not have a notable impact on the toddlers' responses to the fairness task. This outcome aligns with the findings of Ziv and Sommerville (2017), who also reported mixed results regarding the influence of siblings on infants' fairness expectations. Notably, their study found that sibling effects were significant only in older infants (12–15 months), and they did not provide detailed information about the siblings' ages or the nature of their interactions. Our study, like Ziv and Sommerville's (2017), had a small sample size (25 infants with siblings in their study, 20 infants in ours), underscoring the preliminary and exploratory nature of these findings.

While our study like many previous investigations assessed only the presence or absence of siblings, this approach overlooks the complex dynamics that characterize sibling relationships and their potential influence on moral development. Sibling interactions are often emotionally intense and involve frequent negotiations over shared resources, providing daily opportunities for children to practice perspective‐taking, conflict resolution and reciprocity (Dunn 2002). Research indicates that both positive engagement (such as cooperative play and teaching) and antagonism (such as conflict and competition) between siblings contribute to the development of social‐cognitive skills, including theory of mind and empathy, during the preschool years (McAlister and Peterson 2013; Song and Volling 2018). Furthermore, the age gap between siblings, the amount of time spent together, and the specific nature of their shared activities are also likely to be important factors (Brody 2004; Howe et al. 2022). Our study did not capture these nuances, which might explain the lack of association found between sibling presence and fairness expectations.

Future research should move beyond simple sibling counts to examine the frequency, quality, and context of sibling interactions, as well as the roles of age difference and birth order, to more precisely identify how sibling relationships contribute to the early development of fairness understanding. For example, naturalistic observations could assess how siblings resolve conflicts over resources, engage in collaborative play, or teach each other about sharing and turn‐taking (Dunn 2002; McAlister and Peterson 2013). The emotional tone of these interactions, whether characterized by warmth, rivalry, or cooperation may also play a critical role in shaping prosocial and moral development (Song and Volling 2018).

### 8.4 Preschool Attendance and Fairness Expectations

Our results revealed a significant effect of preschool attendance on toddlers' fairness expectations representing a novel contribution to the literature on early moral development. While previous studies have primarily focused on older children with more advanced verbal abilities (Mammen et al. 2019), our findings demonstrate that even at 18 months, children who attend preschool display heightened sensitivity to unfair outcomes in nonverbal fairness tasks. This suggests that the preschool environment plays a unique and influential role in shaping early fairness expectations well before children acquire sophisticated language skills.

To contextualize our findings, it is important to note several distinctive features of the Swedish preschool system. In Sweden, preschool is universally available from age one as part of the welfare system, and most children enroll early (Skolverket 2024). These preschools emphasize social development, maintain low child‐to‐teacher ratios (typically 5–6 children per adult), and provide daily opportunities for peer interaction, cooperation, and conflict resolution. Even toddlers as young as 18 months engage in social conflicts and learn resolution strategies through peer engagement supported by teachers who encourage negotiation, sharing, and turn‐taking (Johansson 2002; Johansson and White 2011). This environment exposes children to a wide range of positive and negative peer exchanges, offering a rich context for learning about fairness, social rules, and the consequences of moral transgressions, even before advanced language skills develop.

Our findings suggest that the mechanism linking preschool attendance to fairness sensitivity likely involves both experiential and verbal components. That is, toddlers may internalize fairness principles through direct participation in resource distribution scenarios, observing peer behaviors, and experiencing the outcomes of fair and unfair actions, potentially in combination with explicit linguistic instruction and adult scaffolding. However, as we did not measure teacher behaviors or their mental state language in this study, the relative contributions of peer experience versus adult guidance remain to be clarified in future research.

However, our study examined preschool attendance as a broad correlate and the specific features of preschool that drive these effects remain to be identified. Future research should move beyond simple attendance and investigate which aspects of the preschool experience are most influential in shaping fairness expectations, such as for instance the frequency and quality of peer conflicts, teacher interventions during disputes, or structured cooperative activities. Observational and longitudinal studies could help clarify whether certain types of social interactions or pedagogical practices within preschools are especially effective in fostering fairness development. Additionally, cross‐cultural comparisons could reveal how variations in preschool structure and philosophy impact the emergence of fairness expectations in early childhood.

### 8.5 Limitations and Future Directions

While our study has provided valuable insights into the development of fairness expectations in toddlers, it is essential to acknowledge and address certain limitations in future research. First, our study's sample size provided a power of 0.75 to detect medium effects in our hierarchical regression models. The non‐significant findings suggest an absence of medium or larger effects, but we acknowledge potential underpowering for smaller effects.

Second, the study's findings are based on a small sample of highly educated Swedish participants, which may limit the generalizability of the results. The conclusions drawn may not necessarily represent the broader population due to potential cultural and contextual factors specific to the Swedish context.

Third, the study relied solely on a single task, the picture‐description task, to measure mental state language. Although informative, this task may not capture the full range of mental state language used by parents in everyday interactions with their children. Future studies could benefit from incorporating more naturalistic observations to assess mental state language across various contexts over time.

Fourth, the study's design did not account for the quality of preschool programs, which can vary widely and may differentially impact moral development. Future research could investigate how specific aspects of preschool education, such as curriculum or teacher‐child interactions, relate to fairness understanding in toddlers.

Future studies should use naturalistic and longitudinal methods to systematically observe sibling conflict resolution, teaching moments, and collaborative play, as these specific forms of interaction may be more predictive of fairness expectations than sibling presence alone (Dunn 2002; Song and Volling 2018). Additionally, examining how parental mediation and discipline strategies moderate the impact of sibling antagonism or cooperation could further clarify the mechanisms at work (Howe et al. 2022).

Additionally, we did not directly code moral talk about right and wrong actions. This type of talk, which explicitly labels behaviors as morally acceptable or unacceptable, may have a different impact on children's moral development compared to mental state talk. While mental state talk may help children understand the intentions and perspectives behind actions, explicit moral talk could more directly shape children's understanding of moral norms and principles. By focusing on mental state talk in a moral context, we aimed to explore the role of perspective‐taking and understanding others' minds in the development of moral reasoning. However, future research should also examine the specific effects of direct moral talk on children's acquisition of moral concepts and their ability to distinguish right from wrong actions.

Our study opens up new avenues for exploring how early social experiences, such as explicit scaffolding or parental praise during play and social interactions, contribute to the development of prosocial behavior and moral reasoning. Longitudinal studies that track these variables over time using new state‐of‐the‐art techniques such as 3D eye‐tracking and emotion AI would be particularly valuable in determining their long‐term impact on children's socio‐moral development.

## 9 Conclusions

We explored the development of fairness expectations in 18‐month‐old toddlers and the influence of social factors on their fairness expectations. Contrary to our expectations, the presence of siblings and parental mental state language during a picture‐description task did not significantly predict toddlers' performance on a fairness task. However, preschool attendance was associated with heightened attention to unfair outcomes, suggesting that peer interactions at preschool could play a more significant role in early fairness expectations than previously understood. These findings indicate that while parental input is valuable for cognitive development, the social dynamics of peer environments like preschools are crucial in shaping toddlers' emerging fairness understanding. Future research should consider the quality of preschool programs to further understand their impact on socio‐moral development.

## Author Contributions

Marek Meristo conceptualized the study, secured funding, designed the methodology, conducted the formal analysis, managed the project, supervised the research, and had primary responsibility for writing the original draft and reviewing and editing the manuscript. Sara Szepanski contributed to data curation and investigation, and participated in reviewing and editing the manuscript. Karin Strid assisted with conceptualizing the study, project administration and supervision, and contributed to writing the original draft and reviewing and editing the manuscript.

## Ethics Statement

The Swedish Ethical Review Authority reviewed and approved the study.

## Funding

This work was supported by the Swedish Research Council (2017‐01076).

## Conflicts of Interest

The authors declare no conflicts of interest.

## Data Availability

All data and materials are available on the Open Science Framework (OSF) at https://osf.io/bz5j9/?view_only=3f272c519433481d9dd7e0204469173a.
